# Completion of Maize Stripe Virus Genome Sequence and Analysis of Diverse Isolates

**DOI:** 10.3389/fmicb.2021.684599

**Published:** 2021-06-14

**Authors:** Stephen Bolus, Kathryn S. Braithwaite, Samuel C. Grinstead, Irazema Fuentes-Bueno, Robert Beiriger, Bryce W. Falk, Dimitre Mollov

**Affiliations:** ^1^National Germplasm Resources Laboratory, United States Department of Agriculture-Agricultural Research Service, Beltsville Agricultural Research Center, Beltsville, MD, United States; ^2^Sugar Research Australia Limited, Indooroopilly, QLD, Australia; ^3^Institute of Food and Agricultural Sciences, University of Florida, Belle Glade, FL, United States; ^4^Department of Plant Pathology, University of California, Davis, Davis, CA, United States

**Keywords:** maize stripe, tenuivirus, diversity, reassortment, *Zea mays*, *Rottboellia cochinchinensis*

## Abstract

Maize stripe virus is a pathogen of corn and sorghum in subtropical and tropical regions worldwide. We used high-throughput sequencing to obtain the complete nucleotide sequence for the reference genome of maize stripe virus and to sequence the genomes of ten additional isolates collected from the United States or Papua New Guinea. Genetically, maize stripe virus is most closely related to rice stripe virus. We completed and characterized the RNA1 sequence for maize stripe virus, which revealed a large open reading frame encoding a putative protein with ovarian tumor-like cysteine protease, endonuclease, and RNA-dependent RNA polymerase domains. Phylogenetic and amino acid identity analyses among geographically diverse isolates revealed evidence for reassortment in RNA3 that was correlated with the absence of RNA5. This study yielded a complete and updated genetic description of the tenuivirus maize stripe virus and provided insight into potential mechanisms underpinning its diversity.

## Introduction

Rice, maize, and sorghum are staple food crops. Diverse plant pathogens can threaten global food security and agricultural economies by infecting these vital crop plants and reducing their marketable yield. *Maize stripe virus* is a tenuivirus species that induces stippling symptoms between leaf veins on corn (*Zea mays* L.), which later can coalesce into continuous chlorotic stripes. Furthermore, infection of young plants often leads to stunting and dramatic “hoja blanca” or white leaf symptoms (Falk and Tsai, 1998). The first scientific reports of maize stripe virus (MSpV) were from Hawaii, Cuba, Trinidad, Mauritius, and East Africa (Storey, 1936). Serological testing of MSpV isolates from the United States (Florida), Venezuela, Peru, Australia, India, Mauritius, Réunion, Thailand, and Taiwan showed that they were all related (Gingery et al., 1979; Greber, 1981; Peterschmitt et al., 1987, 1991; De Doyle et al., 1992; Chen et al., 1993; Sdoodee et al., 1997). Besides infecting corn plants, MSpV isolates have caused disease on sorghum [*Sorghum bicolor* (L.) Moench] in India (Peterschmitt et al., 1991; Srinivas et al., 2014) and itchgrass [*Rottboellia cochinchinensis* (Lour.) Clayton] in the United States (Florida) (Gingery et al., 1981). The host specificity and geographical distribution of MSpV are largely explained by that of its vector, the corn planthopper *Peregrinus maidis* Ashmead, which transmits MSpV in a circulative-propagative manner (Tsai and Zitter, 1982; Nault and Gordon, 1988; Falk and Tsai, 1998; Singh and Seetharama, 2008). Corn planthoppers are also capable of transmitting MSpV transovarially (Tsai and Zitter, 1982).

MSpV is serologically related to the tenuivirus species *Rice stripe virus* (Gingery et al., 1983), which is vectored by the small brown planthopper *Laodelphax striatellus* Fallén. There are reports of rice stripe virus (RSV) infecting maize (Gingery et al., 1983; Bradfute and Tsai, 1990), although its infamy comes from its epidemics on *japonica* cultivars of rice (*Oryza sativa* L.) in Eastern Asia (Wang et al., 2008; Otuka et al., 2010). Both MSpV and RSV are grouped in the genus *Tenuivirus* in the family *Phenuiviridae* (Abudurexiti et al., 2019). Tenuiviruses and the vertebrate-infecting viruses in the genus *Phlebovirus* share conserved complementary RNA end sequences and commonalities in their nucleoprotein, RNA-dependent RNA polymerase (RdRp), and glycoprotein sequences (Falk and Tsai, 1998). However, in contrast to the enveloped virions of phleboviruses, tenuiviruses (from “tenuis,” meaning slender in Latin) are distinguished by their non-enveloped, thread-like ribonucleoprotein particles (Ramírez and Haenni, 1994; Falk and Tsai, 1998). Tenuivirus genomes also differ from those of phelboviruses in that they often have four or five negative and ambisense RNAs (Ramírez and Haenni, 1994; Falk and Tsai, 1998).

The genome of a Florida (United States of America) isolate of MSpV was composed of five RNAs (Falk and Tsai, 1984), and the complete sequences for RNAs 2–5 were determined (Huiet et al., 1991, 1992, 1993; Estabrook et al., 1996). In a separate effort, a partial sequence of RNA1 from an isolate of MSpV from Réunion (France) was determined (Mahmoud et al., 2007). These efforts revealed that RNA1 most likely encodes an RdRp with similarity to that of RSV (Mahmoud et al., 2007). RNA2 is ambisense and encodes p2, a putative membrane-associated protein, on the viral RNA strand and pc2, a putative glyco-polyprotein, on the viral complementary RNA strand (Estabrook et al., 1996). RNA3 and RNA4 are also ambisense with RNA3 encoding p3 and pc3, the nucleocapsid protein (Huiet et al., 1991), and RNA4 encoding p4, the major non-capsid protein, and pc4 (Huiet et al., 1990, 1992). The major non-capsid protein accumulates at very high amounts *in planta*, forming inclusion bodies and needle-shaped crystals that are visible by light microscopy (Bradfute and Tsai, 1990; Falk and Tsai, 1998). RNA5 only encodes pc5, a highly basic, hydrophilic protein of unknown function (Huiet et al., 1993). The intergenic regions in the ambisense RNAs are thought to be important for transcription termination and contain a conserved inverted repeat sequence motif that may form a stem-loop structure (Zhang et al., 2007).

There have been notable advancements in the molecular characterization of several proteins encoded by RSV, the type member of the genus *Tenuivirus*. The p2 and p3 proteins were shown to be silencing suppressors *in planta* (Xiong et al., 2009; Du et al., 2011). The p3 protein also functioned as a silencing suppressor for another tenuivirus, *Rice hoja blanca virus* (Bucher et al., 2003). The glyco-polyprotein pc2 was identified as a helper component for RSV, allowing it to overcome the midgut barriers of its insect vector (Lu G. et al., 2019). In addition, pc4 was recognized as the *in planta* movement protein for RSV (Xiong et al., 2008; Fu et al., 2018).

In this paper, we report the first complete genome sequence for an isolate of MSpV. Using high-throughput sequencing (HTS), we sequenced three additional isolates from *Z. mays* collected in the United States of America. We also identified and sequenced seven isolates of MSpV from *Z. mays* and *R. cochinchinensis* plants collected in Papua New Guinea. We compared these 11 sequenced isolates with other tenuiviruses, other MSpV isolates, and each other to explore patterns underlying the genetic diversity of MSpV.

## Materials and Methods

### Plant Material and RNA Extraction

We obtained an RNA sample originating from the genomic sequencing and characterization work previously performed with a Florida, United States of America (USA) isolate of MSpV (Falk and Tsai, 1984; Huiet et al., 1991, 1992, 1993; Estabrook et al., 1996). We refer to this isolate as MSpV21. In 2019, symptomatic leaves were collected from *Z. mays* (maize) in Palm Beach County, Florida, United States. Symptomatic leaves were also collected in 2019 from *Z. mays* and *R. cochinchinensis* (itchgrass) plants from the Ramu Valley in the Madang Province of Papua New Guinea (PNG) as part of joint Sugar Research Australia and Ramu Agri Industries Limited (RAIL) sugarcane disease surveys. Collected leaves from PNG were stored in tubes containing anhydrous granular calcium chloride (Merck, Darmstadt, Germany) as the drying agent, were treated with 25 or 50 kGy gamma irradiation in Australia, and were forwarded to the United States for further processing. RNA was extracted from the leaf samples using either KingFisher Pure RNA Plant Kit (Thermo Fisher Scientific, Waltham, MA, United States) or RNeasy Plant Mini kit (Qiagen, Hilden, Germany) following the manufacturers’ instructions.

### High Throughput Sequencing

DNase treatment, ribosomal RNA depletion, cDNA synthesis, and library preparation were outsourced (SeqMatic, Fremont, CA, United States). Libraries were sequenced on an Illumina NextSeq 500 platform as 75 single end reads. HTS data were analyzed using CLC Workbench 11–20 (Qiagen).

To quantify the number of reads mapping to each RNA of every MSpV isolate, the reads per kilobase per million reads (RPKM) measurements were calculated by taking the total number of reads mapping to each isolate RNA, dividing by the nucleotide length of the RNA and the total number of sample reads, and finally multiplying by 10^9^ (Wagner et al., 2012). Read mapping was performed using CLC Workbench 11–20 (Qiagen).

### Genome Completion for Isolate MSpV21

To confirm the 5′ and 3′ terminal sequences of RNAs 1–5 from MSpV isolate MSpV21, cDNA was first synthesized from RNA using SuperScript III First-Strand Synthesis System for RT-PCR (Thermo Fisher Scientific) and a universal tenuivirus 5′ and 3′ ends primer Tenui (De Miranda et al., 1994) or a genome-specific primer (Supplementary Table 1). The specific RNA end regions for each RNA molecule were then amplified from cDNA using GoTaq Green Master Mix and protocol, Tenui primer, and genome specific primers (Supplementary Table 1). The PCR products thus obtained were ligated to pGEM-T Easy Vector and cloned in competent *Escherichi coli* JM109 cells using the manufacturer’s kit and protocol (Promega, Madison, WI, United States). At least three clones for each end were selected and sequenced using M13 F and M13 R primers (MCLAB, South San Francisco, CA, United States). Final RNA genome alignments were made using Geneious v. 9 (Biomatters, Auckland, New Zealand) and CLC Workbench 11–20 (Qiagen) software.

### Genome Annotation and Analysis

The assembled genome sequences for all 11 isolates were submitted to the National Center for Biotechnology Information (NCBI)’s GenBank database (Table 1). NCBI’s Conserved Domain-Search tool was used to identify the conserved domains present in the pc1 sequence of MSpV21 (Lu S. et al., 2019), and NCBI’s Open Reading Frame Finder “ORFfinder” was used to identify the coding regions of all the MSpV isolates. The Basic Local Alignment Search Tool (BLAST) from NCBI was used to search for related nucleotide and amino acid sequences and to determine their corresponding percent identities.

**TABLE 1:** Depository information for maize stripe virus isolates.

**Maize stripe virus isolate**	**Host plant**	**Country collected**	RNA1*	RNA2*	RNA3*	RNA4*	RNA5*
MSpV21	Zm	USA	MW328593	MW328594	MW328595	MW328596	MW328597
1704-01	Rc	PNG	MW491852	MW491853	MW491854	MW491855	N/A
1704-02	Rc	PNG	MW491856	MW491857	MW491858	MW491859	N/A
1704-03	Zm	PNG	MW491860	MW491861	MW491862	MW491863	N/A
1704-04	Rc	PNG	MW491864	MW491865	MW491866	MW491867	N/A
1909-05	Zm	PNG	MW491868	MW491869	MW491870	MW491871	N/A
1909-06	Zm	PNG	MW491872	MW491873	MW491874	MW491875	N/A
1909-07	Zm	PNG	MW491876	MW491877	MW491878	MW491879	N/A
2002-04	Zm	USA	MW491839	MW491840	MW491841	MW491842	N/A
2002-07	Zm	USA	MW491843	MW491844	MW491845	MW491846	MW491847
2002-10	Zm	USA	MW491848	MW491849	MW491850	MW491851	N/A

Rc, Rottboellia cochinchinensis; Zm, Zea mays; PNG, Papua New Guinea; USA, United States of America.

*National Center for Biotechnology Information accession numbers.

*N/A, not applicable.*

### Recombination and Phylogenetic Analyses

Alignments of RNAs and encoded proteins were made using the ClustalW method in Molecular Evolutionary Genetics Analysis (MEGA) X under default settings (Kumar et al., 2018). When appropriate, RNA alignments were trimmed at the ends, since the terminal sequences were not determined for all the isolates. Recombination Detection Program v.4.101 (RDP4) (Martin et al., 2015) was used to identify any possible recombinant regions in the individual RNA alignments and was also used to identify any possible RNA reassortments using a concatenated RNA sequence alignment as input. A full exploratory recombination scan was performed after selecting options of linear sequences, 0.05 *P*-value, and Bonferroni correction and selecting the recombination detection methods of RDP, GENECONV, Chimaera, MaxChi, BootScan, SiScan, and 3Seq (Martin et al., 2015). Areas of potential recombination or reassortment were reported only if they were identified by more than four of these selected detection methods under the described significance criteria.

To construct the percent identity matrices, pairwise distances were computed using the Poisson correction model under default settings in MEGA X (Kumar et al., 2018) using selected amino acid alignments as input. Pairwise distances were then converted into percent identities using the following formula: percent identity = 100 – (pairwise distance^∗^100).

To make the phylogenetic trees, selected amino acid and nucleotide alignments were first subjected to model testing in MEGA X (Kumar et al., 2018). Based on the model testing results, the following models were used for the corresponding phylogenies: LG + G + F for RdRp, T92 + G for RNA3 in Figure 3B, GTR + I for RNA1, T92 + G + I for RNA2, HKY + G for RNA3 in Figure 4C, and T92 + I for RNA4. Maximum likelihood phylogenetic trees were constructed using the previously described parameters with 1,000 bootstrap replications and the partial deletion option selected.

**FIGURE 1:**
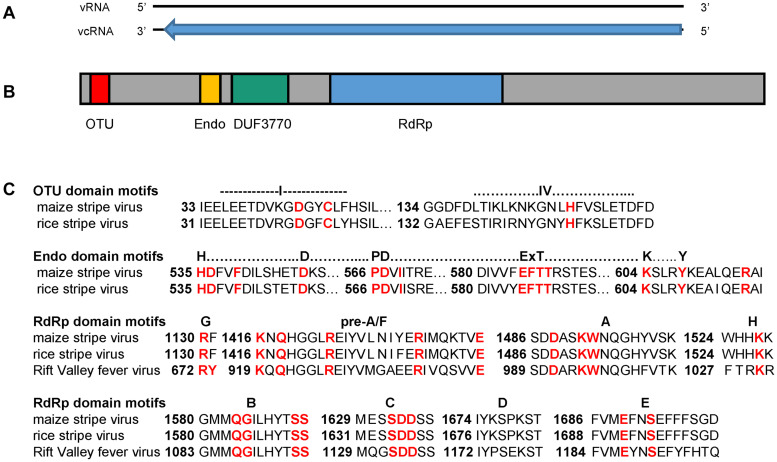
**(A)** Schematic diagram of MSpV21 vcRNA1 at 9,011 nt in length (black bar) with contiguous coding region for pc1 covering nucleotide positions 50–8809 (blue arrow). **(B)** Schematic diagram of MSpV21 pc1 amino acid sequence with conserved domains (colored boxes) identified by the National Center for Biotechnology Information (NCBI) Conserved Domain Search tool (Lu S. et al., 2019) indicated. OTU = Ovarian tumor-like cysteine protease (Accession cl19932/E-value 6.03e-03) (amino acid positions 42-123); Endo = N-terminus bunyavirus endonuclease (Accession cl20011/E-value 2.76e-07) (amino acid positions 512-599); DUF3770 = domain of unknown function found in viruses (Accession cl13978/E-value 9.60e-57) (amino acid positions 648-889); RdRp = Bunyavirus RNA-dependent RNA polymerase (Accession = cl20265/E-value = 0e + 00) (amino acid positions 1,066–1,802) **(C)** Alignment between pc1 sequences of maize stripe virus isolate MSV21 and rice stripe virus (NCBI Reference Sequence NP_620522.1) with focus on the domain regions with conserved and/or essential amino acids marked in red. The RdRp sequence for the phlebovirus Rift Valley fever virus (NCBI Accession ACE78348.1) was also included in the RdRp domain motif analyses.

**FIGURE 2:**
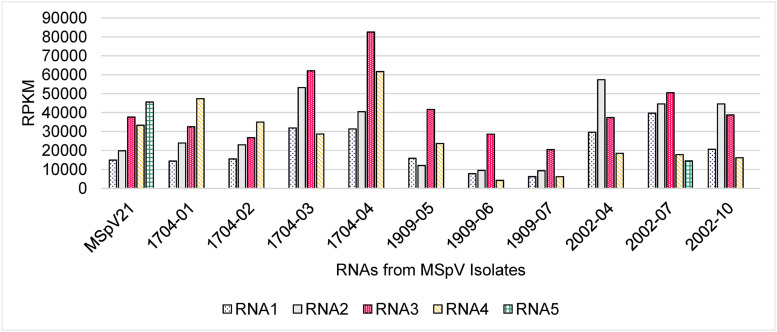
The reads per kilobase per million reads (RPKM) measurements for the maize stripe virus RNA segments as detected by high-throughput sequencing. MSpV, maize stripe virus.

**FIGURE 3:**
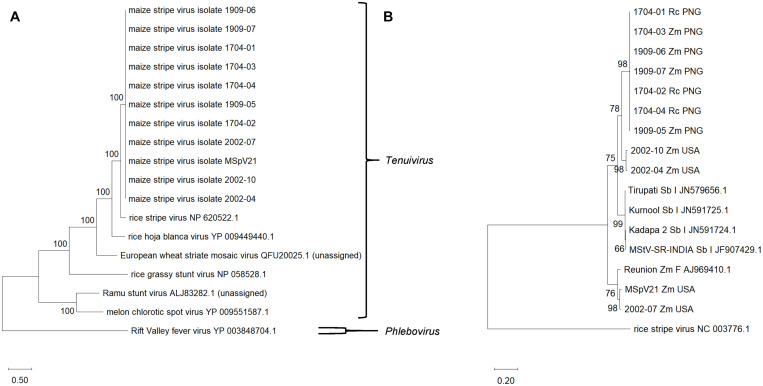
**(A)** Maximum likelihood phylogenetic tree based on RNA-dependent RNA polymerase amino acid sequences from selected tenuiviruses and tenui-like viruses. Rift Valley fever virus, a phlebovirus, was selected as an outgroup. **(B)** Maximum likelihood phylogenetic tree of RNA3 for maize stripe virus isolates. RNA 3 from rice stripe virus was included as an outgroup. The National Center for Biotechnology Information accession and reference sequence numbers are provided to the right of the appropriate names. The numbers at the nodes are bootstrap values, and the scale bar represents the number of substitutions per site. Rc, *Rottboellia cochinchinensis*; Zm, *Zea mays*; Sb, *Sorghum bicolor*; PNG, Papua New Guinea; USA, United States of America; I, India; F, France.

**FIGURE 4:**
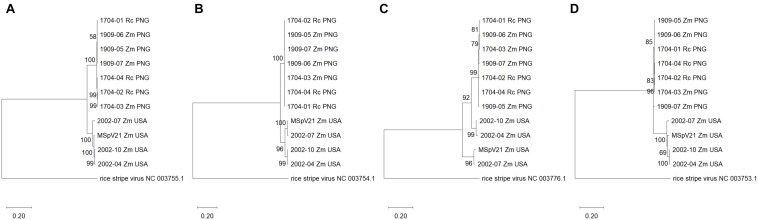
Maximum likelihood phylogenetic trees of each RNA segment for isolates of maize stripe virus. **(A)** RNA1; **(B)** RNA2; **(C)** RNA3; **(D)** RNA4. The respective RNA segments from rice stripe virus were included as outgroups, and the corresponding National Center for Biotechnology Information reference sequence numbers are provided for these. The scale refers to the number of substitutions per site, and the numbers at the nodes are bootstrap values. Rc, *Rottboellia cochinchinensis*; Zm, *Zea mays*; PNG, Papua New Guinea; USA, United States of America.

## Results

### MSpV Genome Completion and Characterization

#### MSpV21 Isolate Genome Completion

Using HTS and completing the ends using Sanger sequencing, we determined the complete genome sequence of MSpV21, an isolate of MSpV that had previously been sequenced, except for RNA1 (Falk and Tsai, 1984; Huiet et al., 1991, 1992, 1993; Estabrook et al., 1996). Our sequences for RNAs 2–5 of MSpV21 were 99–100% identical to those previously deposited in NCBI (Supplementary Table 2). Using ORFfinder (NCBI), we identified the coding regions in our MSpV21 isolate and compared its encoded proteins to those previously deposited in NCBI (Supplementary Table 3). As with the nucleotide sequences, the amino acid sequences were 99–100% identical to the previously deposited sequences (Supplementary Table 3). Excluding other MSpV sequences, BLAST nucleotide and protein searches revealed that MSpV21 RNAs 1–4 and encoding protein sequences were most like corresponding sequences from RSV. RNA5 and pc5 are not present in the RSV genome. These sequences were most like those deposited for tenuivirus *Echinochloa hoja blanca virus* (Supplementary Tables 4, 5).

Tenuiviruses have conserved and complementary end sequences, possibly explaining the circular forms of ribonucleoproteins observed by electron microscopy (Ramírez and Haenni, 1994; Falk and Tsai, 1998). We compiled and aligned the 5′ and 3′ termini of complete, genomic RNAs from our MSpV21, 1704-01, 1704-03, and 2002-07 isolates and compared them to MSpV reference sequences in NCBI (Supplementary Figure 1). The expected conservation and complementarity of end sequences for each MSpV RNA segment was apparent (Supplementary Figure 1). A few exceptions are noted. For RNA1, the sixth nucleotide position from the 3′ end was variable, with 1704-01 and 1704-03 sequences having an A and MSpV21 having a U (Supplementary Figure 1). At the same aligned position for RNA5, there was a U for our MSpV21 sequence, whereas the reference isolate and our 2002-07 sequences had an A at this position (Supplementary Figure 1). We attribute the differences observed at this alignment position to real and/or artifactual genetic variability, since the universal Tenui primer (De Miranda et al., 1994) was used to complete the ends (Supplementary Table 1).

#### RNA1 Characterization

After obtaining the first complete sequence for RNA1 from an isolate of MSpV, we proceeded to characterize the 9,011 nucleotides long RNA1 from MSpV21. ORFfinder (NCBI) identified a long, open reading frame encoding a protein of 2,919 amino acids in the viral complementary strand (Figure 1A). We refer to this putative protein as pc1. RNA1 and pc1 from MSpV are very similar to those of RSV (Toriyama et al., 1994; Supplementary Tables 4, 5). The Conserved Domain-Search tool (NCBI) identified several conserved domains in the pc1 sequence of MSpV21, including ovarian tumor-like cysteine protease (OTU), N-terminus bunyavirus endonuclease (Endo), domain of unknown function found in viruses (DUF3770), and bunyavirus RNA-dependent RNA polymerase (RdRp) (Figure 1B). Further bioinformatic and manual inspection of the OTU domain motifs revealed that the putative OTU in MSpV shared all the amino acids that were previously shown to be conserved and essential for the deubiquitinating enzyme function of OTU domain from RSV (Makarova et al., 2000; Zhao et al., 2020). Closer inspection of the Endo domain also revealed that the H…D…PD…ExT…K…Y motif was conserved between RSV and MSpV, including the essential amino acids for endonuclease activity identified in RSV (Zhao S. et al., 2019; Figure 1C). Analysis of MSpV21’s RdRp domain revealed that the conserved motifs (pre-A/F, A, H, B, C, D, and E) of bunyaviruses” RdRps (Amroun et al., 2017) were present (Figure 1C). For motif G, only the conserved R was present in RSV and MSpV instead of the usual RY (Amroun et al., 2017). Combined, these results indicate that the RdRp of MSpV is very similar to RSV and could be expected to function similarly.

### MSpV Isolate Diversity Analysis

#### Read Mapping to RNAs

Given our HTS methodology and the fact that MSpV is an RNA virus, we could not clearly differentiate between genomic and transcriptomic reads for our MSpV isolates. Nonetheless, we sought to compare the total reads mapping to each RNA across MSpV isolates as expressed in their reads per kilobase per million reads (RPKM) measurements (Figure 2). Of note, RNA5 was only robustly detected in MSpV21 and 2002-07 isolates (Figure 2). Although no uniform pattern of RNA abundance stood out across these sampled isolates, the RPKM measurements between RNAs in any one sample usually did not differ by more than 3.5-fold (Figure 2). These results are like those reported for RSV, where there was at most a 15-fold genomic RNA difference between the four RNA segments *in planta* as measured by absolute real-time quantitative PCR (Zhao W. et al., 2019). The relative abundance of genomic RNAs also varied across time of infection (1–20 days after inoculation) in that study (Zhao W. et al., 2019), possibly explaining the lack of global RNA abundance patterns observed in our isolates.

#### Recombination/Reassortment Analysis

RDP4 (Martin et al., 2015) was used to detect areas of possible recombination in alignments of individual RNAs from all 11 isolates of MSpV sequenced in this study. No areas of recombination that met our significance criteria were detected in the individual RNA 1, 2, 3, and 4 alignments (data not presented). RNA5 was not analyzed for recombination due to the presence of only two sequences from our isolates. To probe for RNA segment reassortment in our 11 isolate sequences, we concatenated RNAs 1–4 for each isolate and aligned them as input for the RDP4 program. RDP4 did detect a region in both 2002-04 (12,354–14,758) and 2002-10 (12,355–14,487) isolates that largely overlapped with the nucleotide positions (12,326–14,638 and 12,326–14,727) of their respective RNA3 sequences in the alignment (Table 2). In fact, the 99% confidence intervals for both regions encompassed the start and stop positions for each isolates’ RNA3 (Table 2). Six out of the seven selected recombination detection methods identified the “recombinant” region at high confidence (probability range 10^–13^–10^–62^) (Table 2). This finding suggests that 2002-04 and 2002-10 isolates could be derived from a reassortment event with RNAs 1, 2, and 4 coming from a parent isolate like MSpV21 and RNA3 from an isolate more like 1704-04.

**TABLE 2:** Potential reassortment regions identified among maize stripe virus isolates using recombination detection program v.4.101 (RDP4).

**Identified “recombinant” isolate**	**Suggested major parent isolate**	**Suggested minor parent isolate**	**“Recombinant” region identified**	**Number of RDP4 methods identifying region**	**Probability range**
2002-04	MSpV21	1704-04	12,354−14,758*	6/7	10^–13^−10^–62^
2002-10	MSpV21	1704-04	12,355−14,487*	6/7	10^–13^−10^–62^

*In the concatenated RNA1-4 sequence alignment, nucleotide positions 12,326−14,638 and 12,326−14,727 corresponded to RNA3 for 2002-04 and 2002-10, respectively. To be noted: the beginning and ending breakpoint 99% confidence intervals of the “recombinant” region for both isolates encompassed RNA3 start and stop nucleotide positions.

#### Phylogenetic Relationships of MSpV Isolates

##### With other tenuiviruses

We assembled a maximum likelihood phylogenetic tree to compare the recently determined MSpV RdRp amino acid sequences with RdRp amino acid sequences from assigned and unassigned tenuiviruses (Figure 3A). The tree was based on 2,017 amino acid positions. The RdRp sequences from MSpV isolates form a clade and are most closely related to the representative RdRp sequence of RSV. The RdRp sequences from melon chlorotic spot virus and Ramu stunt virus formed a clade separate from MSpV, RSV, rice hoja blanca virus, European wheat striate mosaic virus, and rice grassy stunt virus (Figure 3A).

##### With other MSpV isolates

We sought to compare our MSpV isolate sequences to those that are publicly available. The most abundant, complete MSpV RNA sequence that is deposited in GenBank (NCBI) is RNA3. We, therefore, assembled, aligned, and built a maximum likelihood phylogeny based on RNA3 sequences from our MSpV isolates and those that had been deposited in GenBank (NCBI). The resulting tree revealed that our MSpV isolates from PNG form a monophyletic group (Figure 3B). RNA3 sequences from MSpV isolates from *S. bicolor* in India (Srinivas et al., 2014) also formed a monophyletic group (Figure 3B). However, RNA3 sequences from United States split into two separate groups, with 2002-04 and 2002-10 isolates forming a clade with PNG and India isolates and with 2002-07 and MSpV21 forming a separate clade with an isolate of MSpV from Réunion (Mahmoud et al., 2007; Figure 3B). The RNA3 sequence from the Réunion isolate was previously shown to be highly related to the reference MSpV isolate from Florida, United States (MSpV21) (Mahmoud et al., 2007).

##### With each other

We were interested in whether phylograms of individual RNA sequences from each of the MSpV isolates sequenced as part of this study would exhibit the same topologies across the conserved RNAs 1–4. The resulting maximum likelihood phylogenetic trees revealed that for RNAs 1, 2, and 4, the PNG and United States isolates form two distinct, clades (Figures 4A,B,D). However, for RNA3, 2002-04 and 2002-10 United States isolates group apart from other United States isolates and with PNG isolates (Figure 4C), supporting an observation made using comparable parameters in Figure 3B. The data suggest that RNA3 from 2002-04 and 2002-10 may have resulted from an ancestral reassortment event, mirroring the RDP4 analysis results (Table 2). Across the phylogenies, there was no distinct grouping based on host plant (*R. cochinchinensis* and *Z. mays*). In other words, a homogenous virus population appears to infect both plants in PNG.

#### Protein Sequence Identities Among Isolates

**FIGURE 5:**
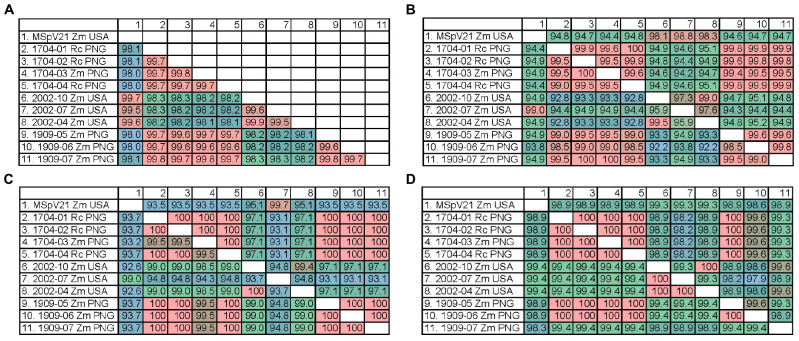
Percent identity matrices of predicted protein sequences from each sequenced isolate of maize stripe virus. **(A)** pc1 from RNA1; **(B)** p2 (bottom) and pc2 (top) from RNA2; **(C)** p3 (bottom) and pc3 (top) from RNA3; **(D)** p4 (bottom) and pc4 (top) from RNA4. Rc, *Rottboellia cochinchinensis*; Zm, *Zea mays*; PNG, Papua New Guinea; USA, United States of America.

We were interested whether the differences observed between isolates in the RNA phylogenies would translate to differences observed at the protein level between isolates. Therefore, we made percent identity matrices for the conserved proteins encoded by RNAs 1–4 of our MSpV isolates (Figure 5). High percent identities (99.5–99.9%) of pc1 between isolates from the same geographic origin were observed, whereas pc1 differed (98.0–98.3% identical) when comparing isolates from distinct regions (Figure 5A). There was also high identity (97.3–100%) among United States and PNG isolates and lower identity between them (94.2–95.2% identical) for pc2. However, p2 percent identity values revealed a third group as 2002-04 and 2002-10 shared lower identity values to both 2002-07 and MSpV21 United States isolates (94.9–95.9%) and PNG isolates (92.2–93.3%) (Figure 5B). The percent identity matrices for p3 and pc3 reflect phylogenetic tree groupings in Figure 4C with sequences from United States isolates 2002-04 and 2002-10 being closer related to PNG isolates (97.1–99.0% identical) than 2002-07 and MSpV21 United States isolates (92.6–95.1% identical) (Figure 5C). Amino acid percent identities overall were high for p4 (lowest 98.3%), and excluding p4 sequences from United States isolate MSpV21 and PNG isolate 1909-07, PNG and United States isolates formed two distinct groups with 100% intragroup identity. The percent identity matrix for pc4 largely reflected geographical origins of the isolates except for the PNG isolate 1909-07, which was more similar (99.6% identical) to pc4 sequences from United States isolates 2002-04 and 2004-10 than those of other United States and PNG isolates (98.9–99.3% identical) (Figure 5D). The pc5 protein sequences from MSpV21 and 2002-07 were compared using BLAST (NCBI). These sequences were 97.6% identical (100% query coverage, *E*-value 0.0). Overall, the data from the protein percent identity analyses support the results from the RNA phylogenetic trees.

## Discussion

We have completed the genome sequence of the reference United States (Florida) isolate of MSpV (MSpV21) and used HTS to determine the genomes of ten additional MSpV isolates from PNG and the United States. There were minor discrepancies between our HTS-derived sequence for the reference MSpV isolate and those previously deposited in NCBI for RNAs 2–5. We largely attribute these differences to the fact that our HTS sequences are derived from consensus sequences of hundreds of thousands of mapped reads and the reference isolate sequences previously deposited in NCBI were mostly derived from several cDNA clones (Huiet et al., 1991, 1992, 1993; Estabrook et al., 1996). In this paper, we also describe, to our knowledge, the first report of MSpV in PNG. We sequenced seven isolates of MSpV from PNG infecting both *R. cochinchinensis* and *Z. mays*. We did not observe evidence of genomic separation by host plant for PNG MSpV isolates, supporting the notion that itchgrass may serve as a reservoir for MSpV as long postulated (Gingery et al., 1981).

The conserved, terminal sequences of MSpV may interact with distinct regions of the RdRp in a pre-initiation configuration, as was shown for La Crosse orthobunyavirus (Gerlach et al., 2015; Amroun et al., 2017). Consensus terminal sequences for RNAs 2–5 of our MSpV genomic RNA sequences matched those for the published MSpV reference isolate (Huiet et al., 1991, 1992, 1993; Estabrook et al., 1996). The first and last 10 nucleotides are largely conserved across RNA segments as also observed for RSV (Takahashi et al., 1990). In addition, the terminal 11–20 nucleotides were conserved within MSpV RNA segments but varied across RNA segments (Takahashi et al., 1990). The genomic MSpV RNA1 consensus 5′ terminal 5′-ACACAAAGUCCAGAGGAAAC-3′ and 3′ terminal 5′-UUUUUCCUCUGACUAUGUGU-3′ sequences are the same as those published for RNA 1 from RSV (Takahashi et al., 1990), except the nucleotide at position 20 of the 5′ end is A for RSV and C for MSpV. Overall, MSpV RNA and protein sequences are most closely related to those of RSV, except RNA5 and pc5, which are absent in sequenced genomes of RSV and are most closely related to those from Echinochloa hoja blanca virus.

We determined and described the first complete RNA1 sequence for a MSpV isolate. Characterization of the genomic sequence for RNA1 of MSpV21 revealed a sequence for pc1 in the viral complementary strand that was similar to that of pc1 from RSV. Besides having a domain with the conserved motifs of bunyavirus RdRps, the pc1 for MSpV also had predicted OTU and Endo domains. Investigation of the motifs in these domains revealed that MSpV has the conserved elements identified in those from RSV and would, therefore, be expected to function similarly (Makarova et al., 2000; Zhao S. et al., 2019; Zhao et al., 2020). The OTU domain in RSV was shown to have deubiquitinating enzyme activity and is suspected to be involved in the autoproteolytic cleavage of pc1 (Zhao et al., 2020). The Endo domain is thought to function in cap-snatching, a function described in some bunyaviruses, where the viral RdRp cleaves capped mRNAs from the host and uses them to prime transcription of its own genes (Amroun et al., 2017; Zhao S. et al., 2019). Evidence of cap-snatching for RSV and MSpV has been previously described (Falk and Tsai, 1998; Liu et al., 2018; Lin et al., 2020).

Recombination appears to be rare in negative-sense, single-stranded RNA viruses; although for those with segmented genomes like influenza A, genetic exchange can still occur through reassortment (Simon-Loriere and Holmes, 2011). We did not find any strong signatures of recombination by RDP4 in individual alignments of our MSpV RNA segments (data not presented). However, in a concatenated RNA1-4 alignment, RDP4 did identify regions corresponding to RNA3 for 2002-04 and 2002-10 MSpV United States isolates that were suggestive of reassortment. This finding was supported by phylogenetic grouping of individual RNA segments of MSpV isolates, where MSpV isolates grouped with high bootstrap support by geographic origin for RNA segments 1, 2, and 4, but for RNA3, 2002-04 and 2002-10, MSpV United States isolates grouped separate from other United States isolates and with PNG isolates. We consider these data as strong evidence that RNA3 from 2002-04 and 2002-10 isolates are derived from an ancestral reassortment event with RNAs 1, 2, and 4 coming from a parent isolate like MSpV21 and RNA3 from an isolate like 1704-04. Differential groupings by RNA segment were also attributed to reassortment for European wheat striate mosaic virus isolates from Northern Europe (Sõmera et al., 2020) and for RSV isolates from Korea (Jonson et al., 2009a,b, 2011).

We herein report the first complete genomes of MSpV isolates that lack RNA5. By comparing RNA3 sequences of MSpV from across the world, there is distinct clustering based on geographic origin and presence/absence of RNA5. The Réunion isolate contained RNA5 based on observed RNA migration sizes (Mahmoud et al., 2007). The Kurnool isolate from India (Srinivas et al., 2014) had a RNA5 sequence deposited in GenBank (NCBI) under the accession number JN626912.1. Previous analysis of a MSpV isolate infecting sorghum in India also revealed the presence of RNA5 by migration size (Peterschmitt et al., 1991). Therefore, three groups are evident by the RNA3 phylogenetic tree: (1) The PNG and United States isolates lacking RNA5, (2) the India isolates infecting sorghum, and (3) the Réunion and United States isolates containing RNA5.

Since the function of pc5 from RNA5 has not been established in tenuiviruses, it is difficult to speculate how some isolates of MSpV accommodate its absence. RSV lacks pc5 and can infect maize (Gingery et al., 1983; Bradfute and Tsai, 1990); therefore, we hypothesize that pc5 does not have a deterministic role in maize infection. It may, however, influence vector infection and transmission efficiency. Differences in MSpV transmission efficiency by its vector *P. maidis* were already noted in a previous study, where *P. maidis* from United States (Hawaii) transmitted MSpV isolates from Costa Rica and Nigeria more efficiently than an isolate from the United States (Florida) (Ammar et al., 1995). Our data indicate that between United States isolates with and without pc5, there were mostly changes in the coding regions of p2, p3, and pc3. Although the exact mechanisms of function for p2 have not been completely characterized, it may function *in planta* for RSV as a weak silencing suppressor by binding to a rice suppressor of gene silencing and targeting a silencing amplification pathway specific to plants (Du et al., 2011). RSV p2 may also promote systemic movement of RSV *in planta* by interacting with fibrillarin (Zheng et al., 2015). The p3 protein from RSV appears to have a more general silencing suppression function through binding of dsRNA (Shen et al., 2010). Indeed, silencing suppression was demonstrated for p3 of rice hoja blanca virus in both plant and insect cells (Hemmes et al., 2007). The pc3 protein is the nucleoprotein, and it has been shown to be expressed in *P. maidis* (Falk et al., 1987). The pc3 protein also colocalized with proteins that play essential roles in the transmission efficiency and transovarial transmission of RSV by its insect vector (Huo et al., 2014; Liu et al., 2015). Based on existing literature on p3 and pc3 and our data on the RNA3 phylogenetic grouping by presence/absence of RNA5, it is tempting to speculate that an ancestral reassortment event of RNA3 in 2002-04 and 2002-10 isolates helped to accommodate their loss of RNA5. Further sampling and sequencing of MSpV isolates worldwide and the molecular characterization of pc5 are needed to clarify the relationship between MSpV core RNAs1-4 and RNA5.

## Data Availability Statement

The datasets presented in this study can be found in online repositories. The names of the repository/repositories and accession number(s) listed in Table 1 can be found below: https://www.ncbi.nlm.nih.gov/genbank/, MW328593; https://www.ncbi.nlm.nih.gov/genbank/, MW328594; https://www.ncbi.nlm.nih.gov/genbank/, MW328595; https://www. ncbi.nlm.nih.gov/genbank/, MW 328596; and https://www.ncbi.nlm.nih.gov/genbank/, MW328597.

## Author Contributions

DM and BF: conceptualization. SB, SG, and IF-B: methodology. SG: software. SB and IF-B: validation. SB, SG, and DM: formal analysis and data curation. SB, SG, DM, IF-B, BF, KB, and RB: investigation. DM, BF, KB, and RB: resources. SB and DM: writing—original draft preparation. SB, DM, BF, SG, KB, RB, and IF-B: writing—review and editing. SB, DM, and BF: visualization. DM: supervision, project administration, and funding acquisition. All authors read and agreed to the published version of the manuscript.

## Conflict of Interest

KB was employed by Sugar Research Australia Limited, Indooroopilly, QLD, Australia. The remaining authors declare that the research was conducted in the absence of any commercial or financial relationships that could be construed as a potential conflict of interest.
