# Supplementary material for: Completion of Maize Stripe Virus Genome Sequence and Analysis of Diverse Isolates
Source: Front Microbiol. 2021 Jun 14;12:684599. doi: 10.3389/fmicb.2021.684599 (PMC8238005; doi:10.3389/fmicb.2021.684599)
Supplement: Supplementary file 1 [file Data_Sheet_1.PDF]

Supplementary Material

Supplementary Table 1. Primers used to verify ends of Maize stripe virus isolate MSpV21

**Primers**	**Sequence (5' to 3')**	**Use**	**Targeted RNA segment**	**Nucleotide positions**
Tenui	CCCGGGCGGCCGCACACAAAGTC	RT-PCR, PCR	All	The 5' and 3' terminal 10 nucleotides
MSpV_RNA1_R1	TCCAATCCAACTGCCTCATC	RT-PCR, PCR	RNA1	316-335
MSpV_RNA1_F1	CTCACGGGAAGACTCCTACTAT	PCR	RNA1	8409-8430
MSpV_RNA1_R2	GCGTGCCATAAAGAGACAGA	RT-PCR, PCR	RNA1	590-609
MSpV_RNA1_R3	TCCAATCCAACTGCCTCATC	RT-PCR, PCR	RNA1	316-335
MSpV_RNA1_R4	CTGGGTTAGCCCTCAATTTCT	RT-PCR, PCR	RNA1	258-278
MSpV_RNA1_R5	AAGCAGTACCCATCTCCTTTC	RT-PCR, PCR	RNA1	172-192
MSpV_RNA1_R6	GTCAGACACTTCCACTGTTGTA	RT-PCR, PCR	RNA1	118-139
MSpV_RNA2_F1	CATCACTAGCCGTACAGGAAAC	RT-PCR, PCR	RNA2	2594-2615
MSpV_RNA2_R1	TCTCTTTGCTTGTGCGAGATTAG	PCR	RNA2	43-65
MSpV_RNA3_R1	AGTTGCTCGCTATGGATTAGTT	RT-PCR, PCR	RNA3	1941-1962
MSpV_RNA3_F1	CCCTTCCAAGTCCCTTCAATAA	PCR	RNA3	457-478
MSpV_RNA3_F2	GGTCCAGATGATGCTGAGATAC	PCR	RNA3	259-280
MSpV_RNA3_F3	GCGACAATACGACCACTCTT	PCR	RNA3	96-115

MSpV_RNA4_F1	CTCTTACAACCATCCCTTGGAG	RT-PCR, PCR	RNA4	381-402
MSpV_RNA4_R1	CTGTGCTAGGTCAAGGAAGATT	RT-PCR, PCR	RNA4	1255-1276
MSpV_RNA4_R2	AAGAGCTTCCCTGAGCTAGA	RT-PCR, PCR	RNA4	1401-1420
MSpV_RNA4_R3	TTGCCTCTGTGACTCTCTCT	PCR	RNA4	182-201
MSpV_RNA4_R4	GAAGGTGCTCCAGAAATGAATG	PCR	RNA4	306-327
MSpV_RNA4_R5	TGTGACTCTCTCTGGTTCCT	PCR	RNA4	175-194
MSpV_RNA4_F2	CTTGACTCCGCTACTTCACAA	PCR	RNA4	563-583
MSpV_RNA4_F3	CAAAGGCAGAGAGTTCAGACA	PCR	RNA4	490-510
MSpV_RNA4_F4	TGAGAGGACGAGCAGATAGTAG	PCR	RNA4	2047-2068
MSpV_RNA4_F5	AAGAGATCCTAAGACCGCAAAG	PCR	RNA4	1685-1706
MSpV_RNA4_F6	CTCTTACAACCATCCCTTGGAG	PCR	RNA4	381-402
MSpV_RNA4_F7	CATTGTCTTGACTCCGCTACTT	PCR	RNA4	557-578
MSpV_RNA5_R1	AGGCCCTGAACCGATGATATAG	RT-PCR, PCR	RNA5	171-192
MSpV_RNA5_F1	GAGCTGTTGGAGAGAAGGATAA	PCR	RNA5	1054-1075
MSpV_RNA5_R2	CAAAGTGCGCCCTATGATAAAC	RT-PCR, PCR	RNA5	444-465
MSpV_RNA5_R3	CCCTTGCTGACGATCTCTTATG	RT-PCR, PCR	RNA5	243-264

Supplementary Table 2. Nucleotide percent identities for our MSpV21 isolate when compared to the published ones using BLASTn[1] from the National Center for Biotechnology Information (NCBI).

**RNA / NCBI Reference Sequence Number**	**Percent Identity**	**Query Coverage**	**Gaps**	**Nucleotide Length (base pairs)**
RNA2 / NC_038751.1	99.82	100%	1*	3338 / 3337
RNA3 / NC_038754.1	99.79	100%	0	2357 / 2357
RNA4 / NC_038752.1	99.28	100%	12*	2223 / 2227
RNA5 / NC_038753.1	99.77	100%	0	1317 / 1317

[1] Nucleotide Basic Local Alignment Search Tool (BLAST)

*There were gaps in RNA 2 and 4 alignments, but they were outside coding regions.

Supplementary Table 3. Amino acid percent identities for proteins encoded by our MSpV21 isolate when compared to the published ones using BLASTp[1] from the National Center for Biotechnology Information (NCBI).

Protein / NCBI Reference Sequence Number	Percent Identity	Query Coverage	Gaps	Protein Length (amino acids)
p2 / YP_009507898.1	99.50	100%	0	201 / 201
pc2 / YP_009507899.1	99.76	100%	0	832 / 832
p3 / YP_009507903.1	100	100%	0	197 / 197
pc3 / YP_009507904.1	99.37	100%	0	316 / 316
p4 / YP_009507900.1	99.43	100%	0	176 / 176
pc4 / YP_009507901.1	100	100%	0	283 / 283
pc5 / YP_009507902.1	99.47	100%	0	375 / 375

[1] Protein Basic Local Alignment Search Tool (BLAST)

Supplementary Table 4. Top match* for maize stripe virus isolate MSpV21 RNA nucleotide sequences in National Center for Biotechnology Information (NCBI)'s nucleotide collection (nt) database as identified by the BLASTn[1,2] tool.

Maize stripe virus RNA	Top BLAST hit* / NCBI Accession or Reference Sequence Number	Percent Identity	Query Coverage	E value
RNA1	Rice stripe virus isolate YCX07 segment RNA1, complete sequence / EU931494.1	75.07	77%	0.0
RNA2	Rice stripe virus isolate DaL08 segment 2, complete sequence / JQ927426.1	73.68	51%	2e-67
RNA3	Rice stripe virus isolate YYAn9 segment 3, complete sequence / KP083237.1	69.74	59%	2e-84
RNA4	Rice stripe virus isolate HuZ10 segment 4, complete sequence / JQ927415.1	72.5	65%	7e-128
RNA5	Echinochloa hoja blanca virus highly basic hydrophilic 44kDa protein (pc5) gene, complete cds / NC_038936.1	70.69	55%	9e-86

[1] Nucleotide Basic Local Alignment Search Tool (BLAST)
[2]search was optimized for 'more dissimilar sequences (discontiguous megablast)'
*Top BLAST hit after other maize stripe virus sequences were removed.

Supplementary Table 5. Top match* for maize stripe virus isolate MSpV21 predicted protein sequences in National Center for Biotechnology Information (NCBI)'s non-redundant protein sequences (nr) database as identified by the BLASTp[1] tool.

**Maize stripe virus protein**	**Top BLAST hit*/ NCBI Accession Number**	**Percent Identity**	**Query Coverage**	**E value**
pc1	RdRP (Rice stripe tenuivirus) / AFM93827.1	73.83	99%	0.0
p2	NS2 protein (Rice stripe tenuivirus) / ACG58372.1	62.12	98%	2e-88
pc2	NSvc2 protein (Rice stripe tenuivirus) / ACG58324.1	53.84	99%	0.0
p3	nonstructural protein (Rice stripe tenuivirus) / CAF04341.1	67.53	98%	3e-99
pc3	CP protein (Rice stripe tenuivirus) / AAX85959.1	65.82	100%	2e-152
p4	disease specific protein (Rice stripe tenuivirus) / CAF04336.1	73.56	98%	3e-90
pc4	NSVC4 protein (Rice stripe tenuivirus) / AAO40772.1	76.79	98%	4e-161
pc5	highly basic hydrophilic 44kDa protein (Echinochloa hoja blanca tenuivirus) / YP009508264.1	55.23	99%	2e-142

[1]Protein Basic Local Alignment Search Tool (BLAST)
*Top BLASTp hit after other maize stripe virus sequences were removed.

	5′ 3′
MSpV21_RNA1	ACACAAAGUCCAGAGGAAAC…UUUUUCCUCUGACUUUGUGU
1704-01_RNA1	ACACAAAGUCCAGAGGAAAC…UUUUUCCUCUGACUAUGUGU
1704-03_RNA1	ACACAAAGUCCAGAGGAAAC…UUUUUCCUCUGACUAUGUGU
NC_038751.1_MSpV_RNA2	ACACAAAGUCCUGGGUAUAA…AUAUUACCCAGACUUUGUGU
MSpV21_RNA2	ACACAAAGUCCUGGGUAUAA…AUAUUACCCAGACUUUGUGU
1704-01_RNA2	ACACAAAGUCCUGGGUAUAA…AUAUUACCCAGACUUUGUGU
1704-03_RNA2	ACACAAAGUCCUGGGUAUAA…AUAUUACCCAGACUUUGUGU
NC_038754.1_MSpV_RNA3	ACACAAAGUCCUGGGUAAAA…CGAUUACCCAGACUUUGUGU
MSpV21_RNA3	ACACAAAGUCCUGGGUAAAA…CGAUUACCCAGACUUUGUGU
1704-01_RNA3	ACACAAAGUCCUGGGUAAAA…CGAUUACCCAGACUUUGUGU
1704-03_RNA3	ACACAAAGUCCUGGGUAAAA…CGAUUACCCAGACUUUGUGU
NC_038752.1_MSpV_RNA4	ACACAAAGUCCAGGGCAUUU…GAUAUGCCCUGACUUUGUGU
MSpV21_RNA4	ACACAAAGUCCAGGGCAUUU…GAUAUGCCCUGACUUUGUGU
1704-01_RNA4	ACACAAAGUCCAGGGCAUUU…GAUAUGCCCUGACUUUGUGU
1704-03_RNA4	ACACAAAGUCCAGGGCAUUU…GAUAUGCCCUGACUUUGUGU
NC_038753.1_MSpV_RNA5	ACACAAAGUCCUUGGCACCA…UUCGUGCCAAGACUAUGUGU
MSpV21_RNA5	ACACAAAGUCCUUGGCACCA…UUCGUGCCAAGACUUUGUGU
2002-07_RNA5	ACACAAAGUCCUUGGCACCA…UUCGUGCCAAGACUAUGUGU

Supplementary Figure 1. Conserved and complementary end sequences of selected, complete genomic RNAs from MSpV isolates compared with the reference MSpV isolate complete genome end sequences. The National Center for Biotechnology Information Reference Sequence numbers for the reference MSpV isolate sequences are provided. Conserved nucleotide sequences are shaded in grey.
